# Clathrin and LRP-1-Independent Constitutive Endocytosis and Recycling of uPAR

**DOI:** 10.1371/journal.pone.0003730

**Published:** 2008-11-14

**Authors:** Katia Cortese, Macarena Sahores, Chris D. Madsen, Carlo Tacchetti, Francesco Blasi

**Affiliations:** 1 Centro di Ricerca MicroSCoBio/IFOM, FIRC Institute of Molecular Oncology, Dipartimento di Medicina Sperimentale, Sezione di Anatomia Umana, Università di Genova, Genova, Italy; 2 Molecular Genetics Unit, Università Vita Salute San Raffaele and IFOM, FIRC Institute of Molecular Oncology, Milano, Italy; Ordway Research Institute, United States of America

## Abstract

**Background:**

The urokinase receptor (uPAR/CD87) is highly expressed in malignant tumours. uPAR, as a GPI anchored protein, is preferentially located at the cell surface, where it interacts with its ligands urokinase (uPA) and the extracellular matrix protein vitronectin, thus promoting plasmin generation, cell-matrix interactions and intracellular signalling events. Interaction with a complex formed by uPA and its inhibitor PAI-1 induces cell surface down regulation and recycling of the receptor via the clathrin-coated pathway, a process dependent on the association to LRP-1.

**Methodology/Principal Findings:**

In this study, we have found that along with the ligand-induced down-regulation, uPAR also internalizes and recycles constitutively through a second pathway that is independent of LRP-1 and clathrin but shares some properties with macropinocytosis. The ligand-independent route is amiloride-sensitive, does not require uPAR partitioning into lipid rafts, is independent of the activity of small GTPases RhoA, Rac1 and Cdc42, and does not require PI3K activity. Constitutively endocytosed uPAR is found in EEA1 positive early/recycling endosomes but does not reach lysosomes in the absence of ligands. Electron microscopy analysis reveals the presence of uPAR in ruffling domains at the cell surface, in macropinosome-like vesicles and in endosomal compartments.

**Conclusions/Significance:**

These results indicate that, in addition to the ligand-induced endocytosis of uPAR, efficient surface expression and membrane trafficking might also be driven by an uncommon macropinocytic mechanism coupled with rapid recycling to the cell surface.

## Introduction

By localizing the proteolytic activity at the plasma membrane and modulating cell adhesion, migration and proliferation, the urokinase receptor (uPAR/CD87) plays an important role in processes such as wound healing, stem cell mobilization, inflammation, HIV-1 replication, tumour progression and metastasis [1]. uPAR is a glycosylphosphatidylinositol (GPI) anchored protein that binds the urokinase-type plasminogen activator (uPA) at the cell surface thus promoting the conversion of plasminogen into plasmin. Plasmin generation triggers the activation of proteolytic cascades that lead to the degradation of the extracellular matrix (ECM). Lacking transmembrane and cytosolic domains, uPAR is unable to transduce signals. It is therefore generally believed that uPAR associates and activates transmembrane receptors on the cell surface. uPAR has been reported to associate to various proteins like integrins, receptor tyrosine kinases (e.g. EGFR) and a G-protein coupled receptor (e.g. FPRL1), thus affecting cell adhesion, migration and proliferation [1]. However, the direct interaction of uPAR with vitronectin, a protein present in the ECM, has been reported to suffice for the induction of at least part of the uPAR-induced cell signalling, resulting in dramatic changes in cell morphology and increased cell motility [2]. As a GPI-anchored protein, uPAR should be found in lipid microdomains and/or clustered within caveolae, a specialized form of lipid rafts [3]. However, careful biochemical measurements have shown that only a fraction of uPAR segregates with detergent resistant membranes (DRM), the rest partitioning with the detergent soluble (DS) membranes [4]–[5]. Lipid rafts partitioning of uPAR is associated with uPAR dimerization and binding to vitronectin, while plasminogen activation by uPAR-bound uPA is independent of its lipid environment [4]. Interestingly, binding of uPA not only promotes plasminogen activation but also regulates the partitioning of uPAR into lipid rafts independently of its catalytic activity, and such localization is important for uPAR-dependent signalling [5]–[6]. Although an appropriate lipid environment seems to be important for some signalling function of uPAR, their role during uPAR endocytosis remains unknown.

Although tethered to the plasma membrane by a GPI anchor, uPAR is efficiently removed from the cell surface via clathrin coated pits and then recycled back, a property dependent upon lateral association with LDL receptor-related protein-1 (LRP-1) or other members of the LDL receptor family. In the proposed model, the specific uPA inhibitor plasminogen activator inhibitor type-1 (PAI-1) binds covalently to uPAR-bound uPA thus inhibiting its enzymatic activity. The ternary uPAR:uPA:PAI-1 complex rapidly associates to LRP-1 and is sequestered in clathrin-coated pits. The ligands are then targeted to the lysosomes for degradation, while the receptors recycle back to the cell surface [7]–[9]. The partitioning of uPAR within specific domains of the plasma membrane that undergo clathrin-independent endocytosis and macropinocytosis (lipid rafts and ruffles/lamellipodia) suggests that other endocytic mechanisms may regulate uPAR trafficking as well. In mammalian cells, even if clathrin-dependent internalization is the best-characterized pathway of endocytosis, several other clathrin-independent pathways coexist in the same cell [10]. Cargo and machinery proteins regulating these alternative pathways are still poorly characterized; however, it is largely accepted that most of them require cholesterol and that the primary transport carriers eventually merge at the level of the early endosome overlapping with other pathways.

We have undertaken a study of uPAR endocytosis utilizing two cell lines. HEK293-uPAR, an uPAR overexpressing highly-motile cell line, does not produce uPA or PAI-1 and hence cannot carry out uPA:PAI-1-triggered, clathrin-dependent endocytosis. The other motile cell line (HT1080, a human fibrosarcoma expressing high levels of uPAR) on the other hand also expresses high levels of uPAR, uPA and PAI-1 and therefore undergoes clathrin-dependent endocytosis of uPAR. Importantly, both cell lines express LRP-1.

In this report, we have established the existence of a second constitutive endocytic route for uPAR internalization. In fact, in addition to the uPA:PAI-1- and LRP-1-mediated route, both cell lines possess an uncommon uPA:PAI-1-independent, lipid rafts-independent but amiloride-sensitive, macropinocytic-like process that is associated with rapid recycling of uPAR to the cell surface. This endocytic mechanism might support the sustained expression of uPAR at its functional sites as well as ensuring the preservation of a constant membrane area.

## Results

### uPAR is found in early and late/lysosomal compartments in the presence of endogenous ligands

**Figure 1:**
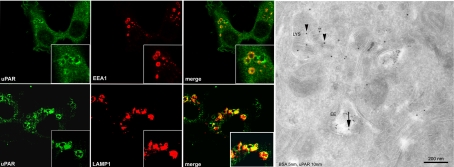
uPAR is present in early endosomes and late endosomes/lysosomes in the presence of endogenous ligands (HT1080 cells). Immunofluorescence of HT1080 cells (left panel) co-stained with anti-uPAR antibody (green) and monoclonal antibody anti-EEA1 (red, top) and monoclonal antibody anti-LAMP1 (red, bottom), respectively, and visualized with confocal laser microscopy. Overlays (merge) show the co-localization of uPAR with EEA1 and LAMP-1 positive membranes (yellow). Scale bars 10 µm. On the right side is shown a representative electron micrograph of uPAR (protein A gold 10 nm) present in early endocytic (full arrow) and lysosomal (arrowhead) compartments previously loaded with BSA 5 nm gold for 2 hours as fluid phase tracer. Scale bar 200 nm.

Formation of the quaternary uPAR:uPA:PAI-1:LRP-1 complex on the cell surface is known to induce clathrin-mediated internalization of the whole uPAR complex, resulting in degradation of the ligands (uPA and PAI-1) and recycling of the receptors (uPAR and LRP-1) [7-8-9]. HT1080 cells express high levels of uPAR, LRP-1, uPA and PAI-1. Therefore, we have assessed the ability of uPAR to internalize in the presence of endogenous ligands by confocal laser microscopy. As shown in **Fig. 1**, HT1080 cells contain uPAR in early endosomal (EEA1 positive) as well as late endosomes/lysosomes (LAMP-1 positive). This observation was confirmed by immuno-electron microscopy using a monoclonal antibody to human uPAR (R3) and 10 nm protein A-gold for detection, which identified uPAR in both early and late endosomal structures loaded with 5 nm BSA-gold for 2 hours at 37°C as endocytic tracer (**Fig. 1**, right). These results indicate that the endogenous uPA:PAI-1 complexes readily internalize and specifically promote partial lysosomal targeting of uPAR in migratory HT1080 cells.

### uPAR is endocytosed in the absence of ligands by a constitutive, uPA:PAI-1 independent pathway

**Figure 2:**
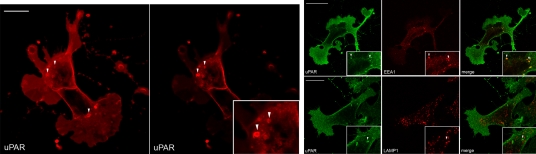
uPAR is found in endocytic compartments in the absence of ligands in HEK293-uPAR cells. Immunofluorescence of HEK293-uPAR cells labeled with a polyclonal anti-uPAR antibody and imaged by confocal microscopy. Image 2 (left), shows the predominant location of uPAR in lamellipodia-like protrusions and in intracellular compartments, including large vesicles (arrowheads). Image 2 (right), shows a different z-axis of the same cell where large putative macropinosomes are evident (arrowheads, inset 3× magnification). Scale bar 10 µm. Image 2B shows HEK293-uPAR co-stained with the early endocytic marker EEA-1 and the late endocytic/lysosomal marker LAMP-1. In these cells, internal uPAR (green) is not present in the lysosomal compartment as shown by the lack of overlap with LAMP-1 (red). On the other hand, intracellular uPAR (green) completely overlapped with EEA-1 positive puncta (arrowheads). Note the large lamellipodia-like protrusions induced by uPAR expression. Scale bar 10 µm. Insets are magnifications of the relevant areas.

In order to study whether uPAR can be internalized independently of its uPA:PAI-1 binding, we took advantage of the human embryonic kidney 293 cell line (HEK293) as these cells do not express endogenous uPAR, uPA nor PAI-1 [4, and our unpublished gene expression profiling, not shown]. Stable uPAR expression in these cells leads to the formation of multiple advancing protrusions that resemble the leading-edge of migratory cells [2]. We therefore analysed the subcellular distribution of uPAR by confocal laser microscopy. Confocal images of HEK293-uPAR cells labelled with anti-uPAR antibodies showed that uPAR was largely located at the cell surface, predominantly in large lamellipodia-like structures and in intracellular vesicles of different size (**Fig. 2 left**). Interestingly, a fraction of uPAR was found in EEA1-positive early endosomal compartments but not in LAMP-1-positive lysosomal compartments (**Fig. 2 right**). These results suggest that in HEK293-uPAR cells the urokinase receptor may be endocytosed also in the absence of uPA and PAI-1, indicating the presence of a constitutive uPAR endocytic pathway. The presence of uPAR in EEA-1-positive membranes in both HEK293-uPAR and HT1080 cells suggests that the two pathways might overlap at the level of the early endosomal system, even though probably distinct mechanisms probably regulates the initial entry. In fact, several evidences show that many clathrin-independent endocytic pathways eventually merge at the level of early (EEA-1 positive) endosome [10]–[12].

**Figure 3:**
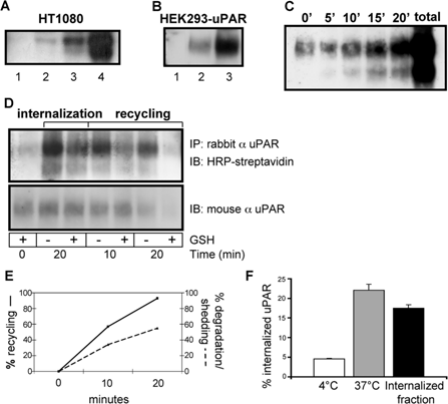
uPAR is constitutively internalized in the absence of ligands. In all cases, the images presented are representative of 3–5 independent experiments. Panel A shows a biotinylation assay performed in HT1080 cells. Cells were acid-washed in order to remove pre-bound ligands, surface biotinylated and then internalization was allowed for 15 minutes at 37°C. Western Blots analysis of cell extracts revealed that uPAR is being internalized in the absence of uPA:PAI-1 in HT1080 (lane 1: background, lane 2: internalized uPAR in the absence of uPA:PAI-1, lane 3: internalized uPAR in the presence of uPA:PAI-1, lane 4: total biotinylated uPAR). Panel B shows the same experiment in HEK293-uPAR cells (Lane 1: background, Lane 2: internalized uPAR in the absence of uPA:PAI-1, Lane 3: total biotinylated uPAR). Panel C shows the biotinylation assay performed in HEK293-uPAR cells, where internalization was allowed for different times, as indicated (total: total biotinylated uPAR). Panel D shows uPAR endocytosis measured by cell cytometry assay in HEK293-uPAR cells. HEK293-uPAR cells were first incubated with anti uPAR polyclonal antibodies bound to Neutravidin-PE for 30 min at 0°C, and then the temperature was raised for 15 min to 37°C. Samples were also incubated at 0°C to account for unspecific binding. After this period, the remaining antibodies at the cell surface were removed by acid washing at 0°C. By cell cytometry we obtained fluorescent intensity values that allow us to calculate the amount of internalized receptor as % of total cell surface uPAR (see materials and methods). White bar: amount of receptor internalized at 4°C. Grey bar: amount of receptor internalized at 37°C. Black bar: actual amount of receptor internalized at 37°C after 15 min, corresponding to 17.5% of cell surface uPAR. Panel E shows the biotinylation assay performed to follow uPAR recycling (see description of the experiment in the text). Lane 1: background. Lane 2: total biotinylated uPAR. Lane 3: internalized uPAR at 37°C (first step). Lane 4 and 6: internalized uPAR (second step) for 10 and 20 minutes 37°C, respectively, without GSH treatment. Lane 5 and 7: internalized uPAR (second step) for 10 and 20 minutes at 37°C, respectively, with GSH treatment. Panel F shows the time-course of uPAR recycling to the cell surface and of its degradation or shedding. The data are expressed in percent of receptors internalized after 20 minutes that is recycled to the plasma membrane (continuous line) or that is lost dring the second incubation (dotted line).

To explore in better detail the basis for the constitutive presence of uPAR in endocytic compartments, we also performed biochemical studies. First, we demonstrated the existence of the constitutive uPAR internalization pathway. HT1080 and HEK293-uPAR cells were acid washed (in order to remove any pre-bound ligands), and then incubated at 4°C with a cross-linking biotin derivative (that does not cross the plasma membrane and can be removed from the cell surface by reduction with reduced glutathione, GSH). The cells were then incubated at 4°C with or without uPA:PAI-1 complexes, and then shifted to 37°C to allow internalization. At this point cells were treated with reduced glutathione to remove the cell surface-associated biotin and lysed. The lysates were immunoprecipitated with anti-uPAR antibodies, blotted and membranes treated with HRP-streptavidin. **Fig. 3A** shows as expected the presence of biotinylated (i.e. endocytosed) uPAR in HT1080 cells both in the absence and presence of exogenous uPA:PAI-1; however, the presence of uPA:PAI-1 increases the level of biotinylated intracellular uPAR. When the same experiment was performed in HEK293-uPAR cells in the absence of exogenous uPA:PAI-1, a fraction of uPAR was also internalized at 37°C, confirming the existence of a constitutive, uPA:PAI-1-independent pathway (**Fig. 3B**). We then performed a time-course analysis of constitutive uPAR endocytosis, and observed that the process was time-dependent (**Fig. 3C**). In this experiment, the presence of the second uPAR band is in agreement with the cleavage of uPAR between domains DI and DII [4].

To more precisely quantify the fraction of internalized uPAR, we developed a flow cytometry assay that evaluates uPAR endocytosis in HEK293-uPAR cells (see Materials and Methods). Briefly, adherent cells were first incubated with anti-uPAR polyclonal antibodies for 30 min on ice, washed to remove the unbound antibody and then transferred to 37°C for 15 min. After this internalization-period, the antibodies remaining at the cell surface were removed by acid washing at 0°C. Flow-cytometry analysis showed that a fraction of cell surface uPAR (17.5%) was internalized during the 37°C incubation (**Fig. 3D**), in agreement with the biochemical data.

Finally, we tested whether constitutively endocytosed uPAR could recycle back to the cell surface. For this purpose, biotinylated HEK293-uPAR cells were incubated at 37°C for 20 minutes, treated with GSH to remove cell-surface biotin, reincubated at 37°C for 10 or 20 minutes, and the amount of cell-surface (recycled) uPAR evaluated after a second GSH treatment by immunoprecipitation with anti-uPAR antibodies and immunoblotting with HRP. The data (**Fig. 3E**) show that the initial GSH treatment removes biotin from the extracellular uPAR (lane 1) and that incubation at 37°C for 20 minutes leads to a substantial GSH-resistance of biotinylated uPAR (i.e. uPAR is internalized) (lane 3). After this step, a second GSH-treatment performed after a econd 37°C incubation, allowed to determine the amount of GSH-sensitive uPAR (i.e. on the cell surface). This increased with the time (lanes 5 and 7), showing that previously endocytosed uPAR was recycled back to the cell surface. The immunoblotting of **Fig. 3E** also show that a substantial amount of uPAR is lost (degraded/shedded) during this second incubation. Figure 3F quantifies both recycling and degradation/shedding.

### The constitutive pathway is clathrin and LRP-1 independent

**Figure 4:**
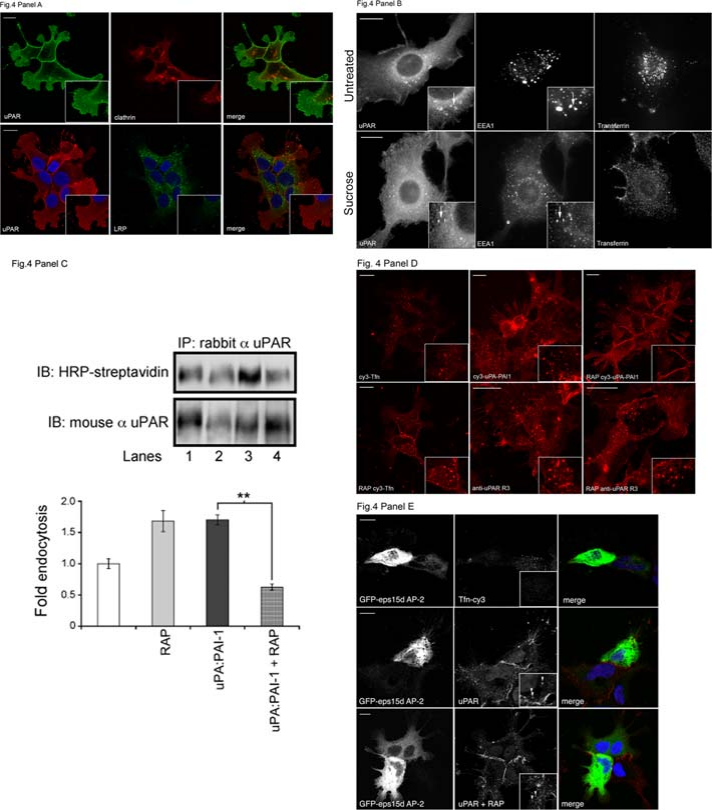
Constitutive endocytosis of uPAR is LRP-1-independent. Panel A: HEK293-uPAR were stained with R3 uPAR (green) and clathrin heavy chain antibodies (red) or with uPAR (red) and LRP-1 antibodies (green). Representative confocal images are displayed. Note that uPAR-rich lamellipodia-like protrusions of HEK293-uPAR cells were largely devoid of clathrin and LRP-1, indicating spatial segregation between uPAR and these endocytic markers. Scale bars 10 µm. Panel B shows acid washed HT1080 cells cultured with Hypertonic Medium to block clathrin-coated pits assembly (0.45 M Sucrose, 45 minutes at 37°C) [13]. Immunofluorescence of uPAR and EEA1 shows co-localization in untreated as well as Sucrose treated cells. The treatment did not change uPAR distribution, although a minimal effect on the organization of the early endosomal compartment was observed. As a control, Transferrin-cy3 was bound to the surface, washed and shifted for 30 minutes at 37°C in untreated and treated cells. As expected, Sucrose treatment significantly perturbed Transferrin entry into the endosomal system. Scale bars 10 µm. Panel C (left) shows a biotinylation assay of uPAR internalization. HEK293-uPAR cells were incubated with 200 nM RAP (Receptor Associated Protein) for 1 hour at 0°C in order to inhibit LRP-1association to uPAR, biotinylated, shifted at 37°C for 15 minutes and GSH-treated. (Lane 1: untreated cells, Lane 2: cells treated with 200 nM RAP, Lane 3: cells treated with 50 nM uPA:PAI-1), Lane 4: cells treated with both 50 nM uPA:PAI-1 and 200 nM RAP). Western Blot analysis indicated that constitutively internalized uPAR is not sensitive to RAP treatment. The right section shows the quantitation of the data obtained by densitometric analysis of the blots. Endocytosis of untreated cells is given the arbitrary value of 1. Panel D shows the uptake of uPAR, uPA-PAI1-cy3 and Transferrin-cy3 in untreated or RAP-treated cells by confocal imaging. HEK293-uPAR cells were incubated with a monoclonal Ab anti-uPAR (R3), uPA-PAI1-cy3 and Transferrin-cy3 for 15 minutes on ice, and then extensively washed before being shifted at 37°C for 15 minutes. Cells were fixed in 3% paraformaldehyde, permeabilized with 0.1% saponin and labelled with anti-mouse cy3 antibody for uPAR detection. Images show that anti-uPAR antibody internalization is not perturbed by RAP treatment (middle panel). On the contrary, uPA-PAI-cy3 endocytosis was strongly inhibited by RAP (right panel). As expected, Transferrin endocytosis was not perturbed by RAP treatment (left panel). Scale bars 10 µm. Panel E shows the effect of the GFP-tagged dominant-negative mutant of eps15 (Eps15 Δ95/295, green) on uPAR ligand-independent uptake by confocal imaging. HEK293-uPAR cells were incubated with anti-uPAR monoclonal antibody and Transferrin-cy3 on ice, washed and then shifted for 15 minutes at 37°C. As expected, cells transfected with the eps15 mutant completely abolished Transferrin uptake (upper panel). Internalization of anti-uPAR antibodies occurred in both cells transfected with the eps15 mutant (middle panel) as well as in cells treated with RAP (lower panel). Images are representative of three different experiments. Scale bars 10 µm.

We next addressed the mechanism regulating the initial entry of uPAR in the absence of uPA:PAI-1 complexes and tested the involvement of the clathrin-dependent pathway. To address this question, we performed confocal double immunofluorescence in HEK293-uPAR cells using antibodies to clathrin and LRP-1 as specific markers for the uPA:PAI-1-dependent, clathrin- and LRP-1-mediated endocytic pathway. As seen in **Fig. 4A**, in these cells uPAR did not co-localize with clathrin or LRP-1at the level of lamellipodia-like membrane domains nor at the cell-cell junctions. In fact, clathrin and LRP-1 were completely excluded from these specialized regions and mainly located in the cell body. The spatial segregation of these endocytic markers and the overall absence of co-localization are suggestive of a potential clathrin- and LRP-1-independent mechanism responsible for constitutive uPAR endocytosis.

Hypertonic treatment of cells is a traditional method used to inhibit clathrin-coated pit formation and clathrin-mediated endocytosis [13]. **Fig. 4B** shows that the intracellular distribution of uPAR in early endocytic (EEA1 positive) components was not affected by incubation of the cells in hypertonic medium (0.45 M Sucrose, 45 minutes at 37°C). This treatment, on the other hand, totally abolished clathrin-mediated endocytosis of Transferrin (**Figure 4B**) and uPA:PAI-1 internalization (not shown), as expected. Overall, these results reveal the existence of a constitutive uPAR internalization route and suggest that it must be different from the ligand-induced clathrin-mediated pathway.

To confirm that LRP-1 is not involved in the ligand-independent uPAR endocytosis, we performed experiments in the presence of the specific LRP-1 antagonist RAP (Receptor-Associated Protein), which inhibits uPA:PAI-1 endocytosis [7], [8]. Indeed, 200 nM RAP did not prevent constitutive internalization of uPAR in HEK293-uPAR cells as demonstrated by cell surface biotinylation and immunoblotting of the cell extracts, as well as by immunofluorescence analysis. As shown in **Fig. 4C** after incubation of cells for 15 minutes at 37°C and removal of the external biotin with glutathione, uPAR was allowed to internalize in the presence of RAP. No inhibition was observed in the level of internalization, compared to controls (lane 1 versus lane 2), indicating that constitutive endocytosis of uPAR is LRP-1-independent. On the contrary, in the presence of uPA:PAI-1, uPAR is internalized at a higher rate, which is inhibited by RAP (compare lanes 3 and 4). Immunofluorescence analysis of HEK293-uPAR cells pre-incubated with a fluorescent monoclonal anti-uPAR antibody on ice and then washed and transferred to 37°C for 10 min also demonstrated internalization of the fluorescent anti-uPAR antibody even in the presence of RAP (**Fig. 4D**). The specificity of RAP to LRP-1 and uPAR internalization was also demonstrated by immunofluorescence since it specifically inhibited uPA:PAI-1-Cy3 but not Transferrin endocytosis (**Fig. 4D**). We conclude that the constitutive internalization of unbound uPAR is independent of LRP-1.

To confirm the clathrin-independence, we blocked clathrin-mediated internalization with a dominant negative, GFP-tagged, mutant of Eps15, EpsΔ95/295, known to impair the interaction with clathrin adaptor proteins such as AP-2, and evaluated the effect on constitutive uPAR endocytosis. We tested by confocal microscopy the ability of anti-uPAR R3 antibody to be internalized in HEK293-uPAR cells transfected with the Eps15 mutant. As shown in **Fig. 4E**, while the internalization of fluorescent transferrin-Cy3 was inhibited in GFP-positive cells, anti-uPAR antibodies were internalized in the presence of the Eps15 mutant as well as of 200 nM RAP. These results support the idea that constitutive uPAR endocytosis is not mediated by LRP-1 or clathrin.

### Lipid rafts are not involved in uPAR endocytosis

**Figure 5:**
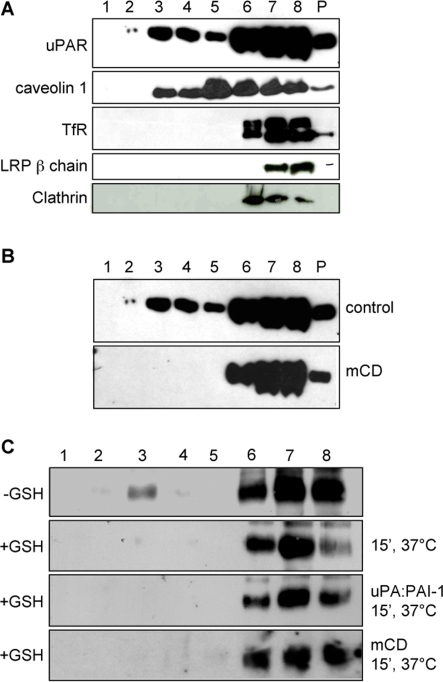
Role of Lipid Rafts in uPA:PAI-1 dependent and ligand-independent uPAR endocytosis. Panel A: Flotation assay of TX-100 extracts of HEK293-uPAR analyzed by immunoblotting. uPAR is located in both detergent-resistant (fraction 2 and 3) but mostly in detergent-soluble fractions of the gradient (lanes 6–8). Caveolin-1 displays a similar distribution, while TfR, LRP-1, β1 integrin and EEA1 are exclusively found in the detergent-soluble fractions. P: pellet. Panel B: uPAR flotation in control and beta-methyl-cyclodextrin (10 mM, 30 minutes at 37°C) treated cells. Under these conditions, uPAR is found only in detergent-soluble fractions (Lanes 6–8). Notice that the flotation in untreated cells is the same as in Panel A. Panel C: flotation analysis of uPAR internalization with cell surface biotinylated HEK293-uPAR cells. UPAR is located in lipid rafts but it is internalized outside of these microdomains. Upper row: cells treated with biotin but not subjected to reduction with GSH, showing the distribution of uPAR present at the cell surface. Second row: uPAR internalized in the absence of ligands (15 min 37°C). Third row: uPA:PAI-1-dependent uPAR internalization. Fourth row: uPAR constitutively internalized in beta-methyl-cyclodextrin treated cells. All images are representative of 3–4 independent experiments.

We then turned our attention to the role of uPAR partitioning into lipid rafts in constitutive endocytosis. Previous work showed that uPAR resides in lipid rafts, and that this localization is important for certain functions of the receptor, such as vitronectin binding and signal transduction [4]. We therefore investigated the role of lipid rafts in uPA:PAI-1-dependent and -independent uPAR endocytosis. We first tested whether uPAR and other relevant proteins were associated with the detergent-resistant membrane (DRM) obtained after solubilization of HEK293-uPAR cells in Triton X-100 at 4°C. Flotation in sucrose density gradients [14] showed (**Fig. 5A**) that only a fraction of uPAR partitioned to DRMs, whereas the largest fraction was found in the detergent-soluble (DS) part of the gradients. These results indicate that a minor fraction of uPAR is enriched in lipid rafts. While caveolin-1 displays a similar distribution, the Transferrin receptor is present only in the DS fractions, as expected. Interestingly, both LRP-1 and clathrin partitioned exclusively to the DS fractions, like β1 integrin and the endocytic marker EEA1 (**Fig. 5B**). In cells depleted of cholesterol with beta-methyl-cyclodextrin (mCD, 10 mM, 30 min. 37°C), uPAR was found only in DS fractions (**Fig. 5B**).

Since uPAR partitions to both detergent-resistant and detergent-soluble fractions, we tested whether partitioning in lipid rafts was necessary for ligand-dependent and -independent uPAR endocytosis. We performed an internalization assay combining cell surface biotinylation with sucrose density gradients (see Materials and Methods). As shown in **Fig. 5C**, biotinylated cell surface uPAR was partially located in the lipid raft fraction, but after 15 min. at 37°C, the (internalized) biotinylated receptor partitioned exclusively to the DS fractions, both in the absence or presence of uPA:PAI-1. Beta-methyl-cyclodextrin (mCD) did not inhibit the uPA:PAI-1-dependent (not shown) nor constitutive uPAR endocytosis (**Fig. 5C**), suggesting that uPAR endocytosis does not require lipid rafts. Altogether, these results indicate that lipid rafts are not involved in either the uPA:PAI-1-dependent or -independent endocytic pathway.

### Ultrastructural characterization of the constitutive pathway

**Figure 6:**
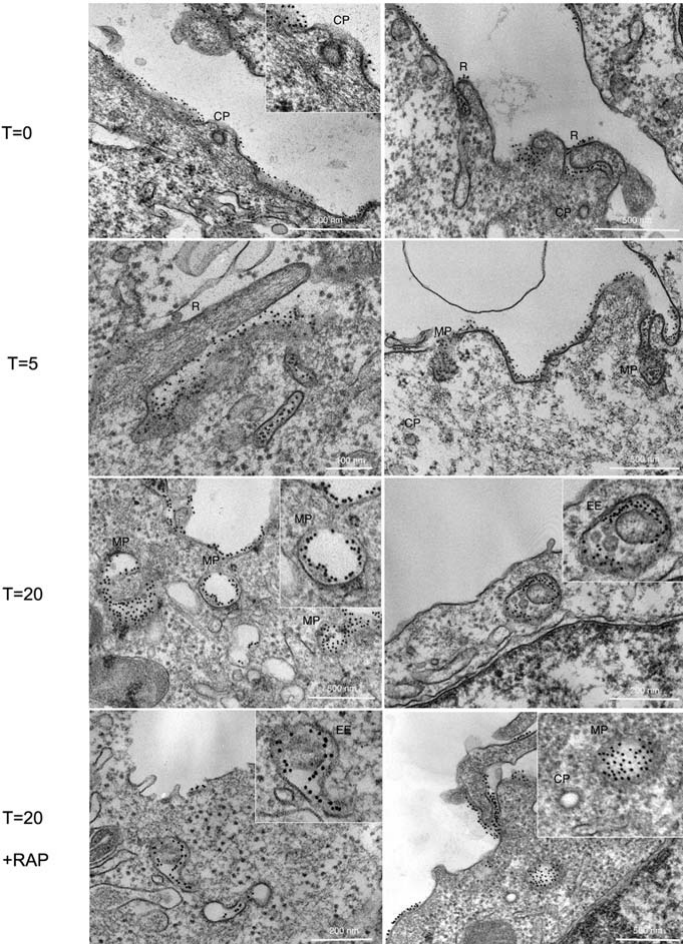
Ultrastructural visualization of uPAR endocytic vesicles. HEK293-uPAR cells were incubated with anti-uPAR monoclonal antibody R3 (that recognizes the D1 extracellular domain) at 4°C for 20 minutes, washed and then probed with protein A gold 10 nm in the absence and in the presence of 200 nM RAP. After extensive washing, cells were warmed in culture medium for 5 to 20 minutes at 37°C to reveal the identity of uPAR primary endocytic vesicles. Samples were then fixed in 2.5% Glutaraldehyde in 0.1 M Sodium Cacodylate buffer and processed for standard EM plastic embedding. At t = 0, uPAR labelling was observed along the plasma membrane, especially in membrane ruffles (R) but excluded from clathrin coated pits (CP). Upon warming at 37°C, uPAR labelling was observed in ruffling regions of the membrane (R), and in clearly large uncoated vesicular profiles of similar morphology to macropinosomes (MP) at t = 5 and t = 20 minutes as well as in early endocytic elements at 20 minutes. uPAR is visible in endocytic vescicles upon 20 minutes internalization in the presence of 200 nM RAP. Note the full macropinosome (MP) and the empty clathrin-coated pit (CP).

GPI-anchored proteins are known to be excluded from clathrin coated pits [15], to associate with caveolae upon antibody cross-linking [16], to be enriched in uncoated tubular carriers termed CLICs/GEEC (early endosomal GPI-enriched comparment) [17] and to be sorted into macropinosomes under certain conditions [18]–[19]. The ultrastructure of the compartment by which uPAR is endocytosed was examined by electron microscopy. We examined the uptake of the monoclonal anti-uPAR Aantibody (R3) in HEK293-uPAR cells and used 10 nm protein A-gold for detection. uPAR and Protein A gold complexes were readily internalized in HEK293-uPAR cells. As shown in **Fig. 6 (t = 0)**, prior to internalization uPAR was evident in ruffling regions (R) of the plasma membrane as well as in distinct non-ruffling domains. Notably, uPAR was largely excluded from clathrin-coated pits (CP) (**t = 0, inset**). Upon 5 minutes at 37°C, uPAR was observed in a variety of structures such as closing ruffles and was clustered within distinct large structures (up to 0.4 µm in diameter) originating from the plasma membrane, with the morphology and size of macropinosomes (MP) (**t = 5, insets**). Consistently with the light microscopy data, uPAR was observed both in large putative macropinosomes (MP) and early endosomes (EE) after 20 minutes at 37°C (**t = 20**). In (**t = 20 RAP**) HEK293-uPAR cells were treated with 200 nM RAP for 1 hour at 37°C prior to the internalization assay in the presence of anti-uPAR R3 antibody and Protein A gold 10 nm. As expected from the RAP-independence of the process (**Fig. 4**), upon 20 minutes of internalization uPAR was found in putative macropinosomes as well as in endosomal structures but not in clathrin coated pits (CP, inset). These results suggest that a macropinocytic mechanism represents the constitutive pathway responsible for a significant fraction of constitutive uPAR endocytosis.

### Constitutive uPAR endocytosis occurs via an amiloride-sensitive endocytic route

**Figure 7:**
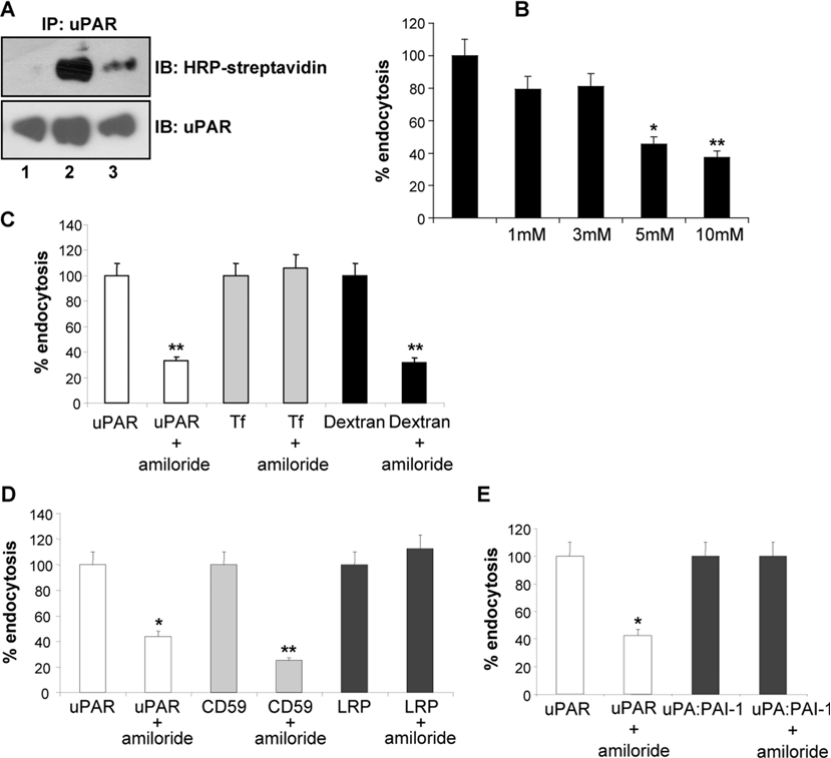
Amiloride inhibits uPAR constitutive uptake in a dose-dependent manner. Panel A. Biotinylation experiments in HEK293-uPAR cells incubated in the absence or presence of amiloride. Lane 1: background, Lane 2: constitutively internalized uPAR, Lane 3: inhibition by amiloride. Panel B: Amiloride inhibits dose-dependently constitutive uPAR endocytosis measured by the cell cytometry assay. Panel C: 5 mM Amiloride specifically inhibits uPAR and 70kDa-Dextran endocytosis, but not Transferrin receptor endocytosis, as observed by the cell cytometry assay. Panel D: Amiloride specifically inhibits uPAR and CD59 but not LRP-1 endocytosis, as observed by the cell cytometry assay. Panel E: Amiloride inhibits uPAR but not uPA:PAI-1 dependent endocytosis, measured by the cell cytometry assay.

The aforementioned results and the localization of uPAR at membrane ruffles and lamellipodia strongly suggest the possible involvement of macropinocytosis [20]–[21]. Sensitivity to inhibitors of Na/H exchangers (e.g. amiloride) is a characteristic of this process [22]–[23]. Consequently we examined the constitutive internalization of uPAR in the presence or absence of amiloride. Using cell surface biotinylation, we observed that uPAR endocytosis was markedly reduced (up to 50%) in a dose-dependent manner by amiloride (**Fig. 7, A–B**). Next, we compared by flow cytometry the effect of amiloride on uPAR, LRP-1, Transferrin, CD59 and dextran endocytosis in HEK293-uPAR cells. Unlike LRP-1 and Transferrin receptors that internalize through clathrin-coated pits, CD59 is a GPI-anchored receptor, like uPAR. Dextran, on the other hand, is a fluid phase marker internalized by macropinocytosis. As shown in **Fig. 7C**, amiloride markedly reduced uPAR and dextran, but not Transferrin endocytosis. Likewise, amiloride significantly inhibited the GPI-anchored CD59 but not LRP-1 endocytosis (**Fig. 7D**). Finally, amiloride did not inhibit uPA:PAI-1 endocytosis (**Fig. 7E**). These experiments demonstrate that uPAR is constitutively internalized by an amiloride-sensitive, macropinocytic mechanism.

### The small GTPases RhoA, Rac-1 and Cdc42 are not involved in the constitutive endocytosis of uPAR

**Figure 8:**
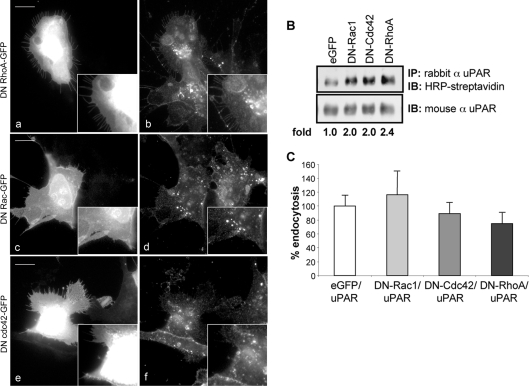
uPA:PAI-1-independent uPAR uptake does not require small GTPases activity. Panel A: constitutive uPAR uptake assessed by immunofluorescence of HEK293-uPAR cells transiently transfected with DNAs encoding GFP tagged mutants of RhoA, Rac1 and Cdc42. A monoclonal anti-uPAR antibody (R3) was bound to the cell surface, cells incubated for 30 minutes at 37°C. Cells were washed, fixed and processed for immunofluorescence. Images (a–b) show uPAR internalization in the presence of dominant negative RhoA, (c–d) the same in the presence of dominant negative Rac1-GFP. The bottom panel (e–f) represents uPAR internalization with dominant negative Cdc42-GFP. None of the mutants inhibited the constitutive endocytic route responsible for uPAR uptake. Data are representative of 2–4 different experiments. Scale bars 10 µm. Panel B: biotinylation assay of ligand-independent uPAR uptake in HEK293-uPAR cells transiently transfected with GFP-tagged dominant-negative mutants of RhoA, Rac-1 and Cdc42. After biotinylation, cells were incubated at 37°C for 15 minutes, lysed, immunoprecipitated with anti-rabbit uPAR antibodies and blotted with mouse monoclonal uPAR antibody or with HRP streptavidin. Western Blot analysis indicates that none of the small GTPase mutants used had a major inhibitory effect on uPAR constitutive uptake. Panel C shows the quantification of uPAR endocytosis by flow cytometry in cells transfected with GFP or small-GTPases-GFP constructs as indicated. Cells were incubated with a polyclonal anti uPAR antibody bound to PE-Neutravidin at 4°C, incubated at 37°C for 15 minutes and then analysed for internalized receptor in GFP expressing cells gating upon GFP-positive cells. These results indicate that small GTPases do not play a role in constitutive uPAR endocytosis.

Rho family GTPases participate in different clathrin-independent endocytic pathways, including macropinocytosis [24]–[25]. Moreover, Rac1 is required for uPAR-induced changes in cell morphology and motility [20]. Therefore, we investigated whether RhoA, Rac1 and Cdc42 were involved in the regulation of the constitutive uPAR internalization. For this purpose we tested by confocal microscopy the ability of anti-uPAR antibodies to be internalized in HEK293-uPAR cells transiently transfected with GFP-tagged dominant negative mutants of RhoA (DN RhoA-GFP), Rac1 (DN-Rac1-GFP) and Cdc42 (DN-Cdc42-GFP). Overexpression of these GDP-bound inactive mutants did not inhibit constitutive uPAR endocytosis (**Fig 8A**). To validate these results, we repeated this experiment using a different approach. Surface biotinylated, mutant GTPases-transfected HEK293-uPAR cells were incubated at 37°C for 15 min. After GSH treatment, the cells were lysed, immunoprecipitated with polyclonal anti-uPAR antibodies and blotted against mouse monoclonal anti-uPAR antibody or directly with HRP-streptavidin. Again, the presence of the dominant negative forms of RhoA, Rac1 or Cdc42 had no major effect on constitutive uPAR endocytosis (**Fig. 8B**). Finally, we confirmed these results by flow cytometry evaluating the internalization of fluorescent anti-uPAR antibodies in HEK293-uPAR cells transfected with the GFP-tagged dominant negative mutants of RhoA, Cdc42 and Rac1. As shown in **Fig. 8C**, GFP-positive cells, transfected with either control GFP or GFP-tagged dominant negative constructs, were equally able to internalize fluorescent anti-uPAR antibody, confirming that Rac1, RhoA and Cdc42 activities are not required for constitutive uPAR endocytosis. In summary, these results suggest that uPAR endocytosis employs an amiloride-sensitive and small GTPase-independent internalization mechanism, possibly a related form of macropinocytosis.

### PI3K activity is not required for uPAR constitutive endocytosis

**Figure 9:**
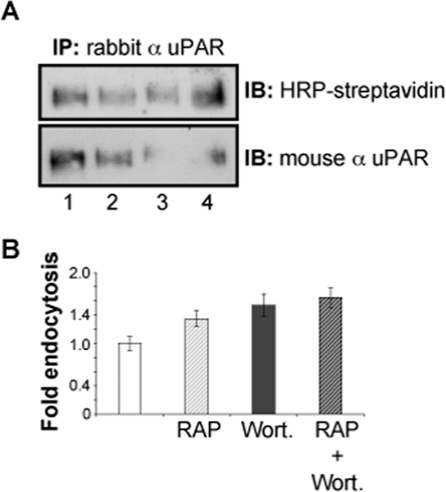
uPAR constitutive endocytosis does not require PI3K activity. Panel A: Biotinylation experiments in HEK293-uPAR cells incubated in the absence or presence of Wortmannin and RAP. Lane 1: no treatment, Lane 2: 200 nM RAP, Lane 3: 300 nM Wortmannin, Lane 4: Wortmannin and RAP. Wortmannin and RAP do not inhibit constitutive uPAR endocytosis after 15 min at 37°C. Panel B: densitometric analysis of the immunoblots of Panel A.

PI3K regulates macropinocytosis by inducing actin ruffles [26]. uPAR internalization assays by cell biotinylation were performed in HEK293-uPAR cells treated with the PI3K inhibitor Wortmannin (300 nM, 1 hour) in order to test the requirement of this kinase in uPAR constitutive macropinocytic uptake. Interestingly, we observed that inhibition of PI3K with Wortmannin slightly increased uPAR constitutive internalization. The effect was even more evident in the presence of both RAP and Wortmannin (**Fig. 9A–B**). These results indicate that the constitutive uptake of uPAR does not require PI3K activity. Taking these results together, we defined the uPAR constitutive internalization as a mechanism that shares the amiloride-sensitivity of macropinocytosis but does not require described factors for macropinocytic processes. We presume that the observed stimulation of uPAR endocytosis when PI3K and small GTPases function were perturbed is due to a general induction of alternative/compensatory pathways in order to maintain cell viability under stressing conditions. This concept agrees with recent views that cells might sort specific cargo in alternative pathways, under different conditions [27].

## Discussion

Endocytosis controls the cellular response to ligands by targeting the ligands for degradation in the lysosome. On the other hand, recycling of the receptors allows the re-stimulation of a signalling pathway. It has been established that LRP-1 mediates the clearance of uPA:PAI-1 complexes from the extracellular milieu and that this process is accompanied by concomitant internalization of uPAR ro which it is bound via uPA [8]–[9]. Internalization of uPAR after binding of the uPA:PAI-1 complex allows to attenuate the extracellular proteolysis (by degradation of the ligand and its inhibitor) and to reposition the receptor at the cell surface after recycling [8]–[11]. Despite the fact that uPA:PAI-1 mediated endocytosis via clathrin-coated pits is largely accepted to be responsible for the removal of uPAR from the plasma membrane, a clathrin-independent and RAP insensitive internalization route for uPA:PAI-1 occupied uPAR has been reported in non-polarized MDCK cells, indicating that uPAR might be internalized also by other mechanisms [28]. Constitutive ligand-independent uPAR endocytosis does not appear to be due to overexpression as it is observed in naturally expressing, non transfected, HT1080 cells (**Figure 1**), in Hela cells and in different HEK293-uPAR clones expressing lower level of uPAR (not shown).

In this paper we have described a new pathway responsible for the constitutive endocytosis of uPAR in HEK293-uPAR in the absence of uPA and PAI-1. This unusual pathway was associated with rapid recycling of the receptor to the cell surface and did not require ligand binding and LRP-1 association nor the activity of regulatory proteins such as members of the Rho GTPases and PI3K, usually involved in macropinocytosis and in promoting cell motility. Biochemical and immunofluorescence studies provided clear evidence that constitutive uPAR endocytosis did not require LRP-1 association since it is insensitive to RAP and does not employ commonly used endocytic routes that are dependent on clathrin, as demonstrated by the use of hypertonic medium and the eps15 dominant negative mutant (Fig. 4). Many clathrin-independent routes require clustering of receptors, including GPI-anchored receptors, in specific cholesterol-rich sub-domains of the plasma membrane termed lipid rafts [10] and cholesterol perturbation significantly affects their endocytosis [17]. However, rare clathrin-independent and lipid rafts-independent routes have also been reported [29]. Despite the fact that specific lipid environment is instrumental for some functions of uPAR [4]–[5], flotation analyses combined with internalization assays clearly showed that endocytosed uPAR did not partition in low density fractions of sucrose gradients, and lipid-rafts disruption by methyl-β-cyclodextrin did not affect either uPA-PA1-dependent or the constitutive uPAR endocytosis.

Macropinocytosis is a form of bulk uptake of fluid and cargo into cytoplasmic vacuoles, called macropinosomes. This process requires extensive plasma membrane reorganization, actin remodelling as well as the coordinated activity of several regulatory proteins. Macropinocytosis is constitutive in some cell types (e.g. dendritic cells and macrophages) but it is also prominent in cells stimulated with growth factors (such as EGF) [30]–[34]. The dynamics of this pathway is still poorly understood, however recent studies support the concept of regulated bidirectional trafficking of macropinosomes, possibly a compensatory mechanism to replete the surface area lost during the endocytic process [35]. Macropinocytosis has been reported to account for the constitutive and LPS-stimulated intracellular sorting of the GPI-anchored CD14 in promonocytic THP-1 cells [18]. Another study reported a direct role for GPI-anchored proteins-enriched macropinosomes in Brucella infection of macrophages. Removal of GPI-anchored proteins from macrophages dramatically inhibited macropinocytosis and infection, suggesting a direct role of GPI-anchored proteins in modulating macropinosome function [19].

The only known mechanism that we could partially tie with the constitutive uPAR endocytosis is macropinocytosis. Our best evidence that uPAR is constitutively endocytosed via macropinocytosis comes from the ultrastructural analysis (Fig. 6). Endocytosed uPAR was found in large structures originated from the plasma membrane with the morphology and size of macropinosomes. Additional evidence in this direction is that uPAR endocytosis is sensitive to amiloride, an extremely potent inhibitor of macropinocytosis [22]. In spite of its effectiveness, the use of amiloride to define uptake of cargo via macropinocytosis [23]–[29] has been recently criticized since amiloride also inhibits albumin uptake in kidney epithelial cells [38]–[39] and alters the cellular pH in cultured cells [40].

Constitutive internalization of uPAR, CD59 and the uptake of a 70kDa-dextran, but not of transferrin and LRP-1 was dramatically perturbed by amiloride treatment, thus falling in the fluid-phase, macropinocytosis category. We have also demonstrated that uPAR is eventually sorted to EEA1-positive endosomes. Upon endocytosis, early uPAR-enriched macropinosomes might acquire regulatory proteins such as EEA1 and fuse with pre-existing early/sorting endosomes. Interestingly, both the constitutive and ligand-dependent pathways provide trafficking via EEA1-positive early endosomal compartments and efficient recycling [8], but the latter mechanism requires clathrin and LRP-1. Since the uPA:PAI-1-mediated route regulates important extracellular uPAR functions, we speculate that also the constitutive pathway supports similar functions under different conditions, i.e. in the absence of ligands/inhibitors.

The interaction of uPAR with vitronectin induces strong cell adhesion [2]. uPAR binds the matrix protein vitronectin as a dimer [4] in the basal membrane of the cell. This dimerization is dissociated by uPA:PAI-1, leaving only monomers at the basal membrane [4]. Possibly the constitutive internalization regulates the constitutive adhesion/release of uPAR/Vn adhesion which is an important step during cell migration. Obviously, this mechanism might operate in both ligand-dependent and independent conditions, i.e. in parallel with a Clathrin/LRP-1-mediated or in a macropinocytosis-type turnover of uPAR/vitronectin adhesion.

Therefore, EM morphology and amiloride sensitivity suggest that constitutive uPAR endocytosis would follow a macropinocytic route. However, classical macropinocytosis has been shown to be lipid rafts dependent and to require Rac-1 activity and upstream effectors such as PI3K, Ras and PLC. From our investigations, neither Rac-1 nor PI3K appear to be required for uPAR internalization. In fact, even though lamellipodia formation in uPAR overexpressing cells is documented to require Rac-1 activity [20], we found that the constitutive internalization of uPAR was Rac-1 independent. Macropinocytic mechanisms that do not require Rac-1 and actin dependent membrane ruffling have been reported [22]–[36], suggesting that ruffling and macropinocytosis are not entirely dependent on Rac1 activity. In addition, since all these factors are also considerably involved in promoting cell motility, it is possible that the uPAR constitutive pathway is not related to cell motility. Potentially, the insensitivity to Rac-1 inhibition might also be explained as the result of a association between uPAR and the EGF receptor [37]. In fact, uPAR association with the EGF receptor activates ERK signalling pathway independently of Rac-1.

The link between growth factors stimulation and macropinocytosis is well documented [30]–[33]. Growth factors stimulation has been recently associated to a second type of ruffles termed circular dorsal ruffles (CDR or dorsal waves). Whereas lamellipodia are associated with cell motility, growth factor-induced circular ruffles are involved in macropinocytosis and in three-dimensional migration [33]–[34]. Whether uPAR expression induces dorsal ruffles is currently unknown and would be of significant interest to further elucidate the basis of the macropinocytic mechanism.

PI3K inhibitors such as the fungal metabolite Wortmannin dramatically affect fluid phase uptake and early endosome homotypic fusion. In macrophages, wortmannin also inhibits macropinocytosis reducing the closure of ruffles rather than the formation of protrusions. Our results, however, indicate that PI3K activity was not required for the constitutive sorting of uPAR (Fig. 9).

Many receptors, like those necessary for maintaining cellular homeostasis, are recycled back to the cell surface, whereas receptors whose signals need to be attenuated are delivered to the lysosomal compartment. We have monitored the endocytic compartments in which uPAR travels after endocytosis. Immunofluorescence studies using early endocytic and lysosomal markers revealed that uPAR is distributed among the overall endocytic pathway in cells (HT1080) producing the natural ligands of uPAR, however in HEK293-uPAR cells uPAR failed to reach lysosomes in the absence of these ligands (Fig. 1A, Panel B). The association of uPAR, in the absence of ligands, with early endosomal compartments, supports its rapid recycling, possibly to ensure efficient membrane retrieval and its constant exposure at the leading-edge. We have demonstrated biochemically and through flow cytometry that once uPAR is sorted into macropinosome vesicles it is rapidly recycled back to the cell surface (**Figure 3, E–F**).

In conclusion, we have described a novel mechanism supporting constitutive endocytosis and recycling of uPAR that shares ultrastructural properties and amiloride sensitivity with macropinocytosis, while is clearly distinct from the clathrin-mediated uPA:PAI-1 endocytic pathway. In this respect, uPAR might represent a model for the constitutive internalization of GPI-anchored receptors.

## Materials and Methods

### Cell Culture

HT1080 cells and HEK293 cells stably transfected with human uPAR were cultured in Dulbecco's Modified Eagle Medium supplemented with penicillin (100 U/ml), streptomycin (100 U/ml), glutamine and 10% fetal bovine serum at 37°C in 5% CO_2_. HEK293-uPAR cells were maintained in 0,2 mg/ml G418. Techniques have been described [2].

### Materials

Cell culture media and supplements were purchase from Gibco-BRL (Carlsbad, CA, USA) and plastic ware was from Costar (Cambridge, MA, USA). Triton X-100, methyl-β-cyclodextrin, amiloride hydrochloride hydrate, Sucrose, Wortmannin and general laboratory chemicals were from Sigma-Aldrich (St. Louis, MO, USA). Protein G-Sepharose beads and reduced glutathione (GSH) were from Amersham Pharmacia Biotech (Buckinghamshire, UK). Secondary HRP-conjugated antibodies were from Amersham Pharmacia Biotech (Buckinghamshire, UK). PVDF membranes were from Millipore (MA, USA). HRP-conjugated streptavidin and the transfection reagent Fugene 6 were from Roche (Basel, Switzerland). Chemoluminescent substrate and EZ-linked Sulfo-NHS-SS-Biotin were from Pierce (IL, USA). Clinical grade uPA was obtained from Crinos (Italy). The polyclonal anti-uPAR antibodies as well cy3-conjugated uPA and uPA-PAI1 were prepared as described below. The LRP-1 agonist RAP (Receptor Associated Protein) and the monoclonal antibodies anti-515 KDa chain (8G1) were a generous gift from D. K. Strickland (Surgery and Physiology, University of Maryland Medical School, Rockville). The R2, R3 and R4 anti-uPAR antibodies were kindly provided by Dr Gunilla Høyer-Hansen (Finsen Laboratory, Copenhagen, Denmark). The monoclonal antibody X22 (ab2731) recognising clathrin-heavy chain was purchased from Abcam (Cambridge, UK). The monoclonal antibodies to caveolin-1 and Transferrin Receptor were purchased from Zymed Laboratories (San Francisco, CA, USA). The rabbit polyclonal antibodies to Caveolin-1 were obtained from Santa Cruz Biotechnology Inc. (Santa Cruz, CA, USA). Monoclonal antibody to EEA-1 was a gift from C.Valetti (MicroScoBio/IFOM Genova). The monoclonal anti LAMP-1 antibodies (H4A3) were obtained from Hybridoma Bank (Iowa City, IA, USA). The monoclonal antibodies to CD59 were purchased from Serotec (Oxford, UK). The donkey anti-rabbit and donkey anti-mouse secondary antibodies labeled with fluoresceine and rhodamine were purchased from Jackson (Westgrove, PA, USA). Anti-rabbit and anti-mouse Alexa 488 and Alexa 546 for confocal experiments, as well as Phycoerythrin-Neutravidin, cy3-labeled Transferrin and Oregon Green 70000 MW-Dextran were purchased from Molecular Probes (Paisley, UK). BSA conjugated to 5-nm gold particles and protein A gold conjugates were purchased from J.W. Slot (Utrecht Medical School, Utrecht, The Netherlands). The wt and dominant negative, GFP-tagged versions of Eps15 (pEGFP eps15 wt and pEGFP eps15ΔAP2, respectively) were a generous gift from Simona Polo, (IFOM, Milano, Italy). Dominant negative, GFP tagged versions of the small GTPases Cdc42 (pcGFP.cdc42N17), RhoA (pcGFP.RhoAV14), Rac1 (pcGFP.RacN17) were a kind gift of Daniela Talarico (San Raffaele Scientific Institute, Milano, Italy).

### uPA:PAI-1 complexes

In order to study LRP-1-mediated uPAR internalization, uPA:PAI-1 complexes were prepared. First, uPA and PAI-1 activity was tested. Caseinolytic zymography was done in order to test uPA activity, by preparing agarose plates with 1% agarose, 2,5% non fat dry milk, 0,1 M Tris and 40 µg/mL plasminogen. After solidification, holes were made in the plate in order to load uPA. After 2 h at 37°C, clear plaques were seen in those holes where uPA activated plasminogen. To test PAI-1 activity, reverse caseinolytic zymography was employed. Agarose plates were prepared with 0,8% agarose, 0,8% non-fat dry milk, 0,1 M Tris, 13 µg/mL plasminogen and 90 µg/mL uPA. After incubation 3h at 37°C, white plaques were seen only where PAI-1 inhibited uPA activity. Then, uPA:PAI-1 complexes were formed as previously described [43], by combining uPA and a 5-fold molar excess of PAI-1 overnight at 37°C , and its formation was checked by 10% SDS-PAGE. Free uPA retaining enzymatic activity was removed by affinity chromatography in Benzamidine Sepharose 6B. The complexes were conjugated with cy3 dye according to the manufacturers (Molecular Probes, Paisley, UK).

### Immunofluorescence analysis and transfection

HT1080 cells and HEK293-uPAR cells were first quickly washed in PBS and fixed in 3% paraformaldeyde in PBS at room temperature, quenched with 30 mM NH_4_Cl, permeabilized with 0,1% Saponin and incubated for 20 minutes in PBS/Saponin 0,1% with a mix containing anti uPAR polyclonal antibodies or the monoclonal antibodies (R4 or R3) and the antibodies to EEA-1, LAMP-1, LRP-1, and clathrin markers. Fluorescent signal was detected by the use of Alexa-conjugated 488 or 546 secondary antibodies. For internalization of uPA-PAI1-cy3 and Transferrin-cy3 (5 µg/ml) and the monoclonal antibody R3 (anti human uPAR), cells grown overnight on glass coverslips, where first washed in HBSS medium and incubated with the uPA-PAI1-cy3 complex or Transferrin-cy3 in the same medium for 10 minutes at 0°C. Cells were incubated at 37°C for 10 or 15 minutes to allow internalization. The excess of reagents was removed by extensive washing with HBSS and then cells were processed as previously described. For experiments with RhoA, Rac1 and cdc42 GDP-bound mutants, HEK293-uPAR cells were cultured on glass coverslips and transiently transfected using Fugene 6 (Roche) for 24 hours with 1 µg of the indicated DNA construct. To analyze uPAR trafficking under these conditions, transfected cells were incubated with anti-uPAR mAb R3 for 30 minutes on ice, washed twice and incubated at 37°C for 15 minutes. Cells were then fixed, permeabilized and labelled with anti-mouse cy3 conjugated antibody. To perturb clathrin-mediated internalization we used Hypertonic Treament (0.45 M Sucrose, 45 minutes at 37°C) [13] and the GFP-tagged eps15 mutant EpsΔ95/295 in HEK293-uPAR cells. Cells were transfected as mentioned above. To analyze uPAR trafficking under clathrin inhibition, we incubated cells with anti-uPAR mAb R3 for 30 minutes on ice, washed twice and incubated at 37°C for 15 minutes. Transferrin-cy3 was used as a control to test the efficacy of the mutant. Cells were then fixed, permeabilized and labelled with anti-mouse cy3 conjugated antibody to reveal uPAR and analyzed. Confocal images were acquired using a Leica TCS SP2 confocal microscope (Leica Microsystems, Heidelberg, Germany). Image analyses were performed using Adobe Photoshop version CS. For each condition, at least 10–20 cells were imaged.

### Protein distribution in sucrose density gradients

Subconfluent HEK 293-uPAR cells were washed twice with cold PBS, collected by scraping and centrifuged 5 min at 1200×g. Cells were lysed for 30 min on ice in ice-cold buffer A (150 mM NaCl, 2 mM EDTA, 50 mM Tris-HCl, pH 7.5) containing 1% TX-100 and a cocktail of proteinase inhibitors (Complete, Roche, Basel, Switzerland). Cell lysates were centrifuged at 4°C for 5 min. at 1200×g. One ml of this postnuclear supernatant was brought to 40% sucrose using 80% sucrose in buffer A plus detergent, placed at the bottom of a ultracentrifuge tube (Beckman Instruments Inc, Fullerton, CA, USA) and overlaid with 4 ml of 30% sucrose and 2 ml of 5% sucrose in buffer A plus detergent, and filled up with 4 ml of buffer alone. Samples were centrifuged at 0°C for 17 h at 39,000 rpm (Beckman ultracentrifuge rotor SW41Ti). After centrifugation, 1 ml fractions were collected from the top to the bottom of the gradient using a pipette. Eight fractions were collected. Fractions 2 and 3 were determined to correspond to detergent-resistance membrane (DRM) and fractions 7 and 8 to detergent soluble membranes (DS). Then the samples were analyzed by SDS-PAGE followed by immunoblotting for the proteins of interest.

### Methyl-β-cyclodextrin (mCD) treatment

For biochemical analyses, HT1080 and HEK293-uPAR transfected cells were incubated with 10 mM of mCD for 30 min at 37°C. After the treatment, sucrose density gradients were performed as previously described [14].

### Distribution of uPAR in sucrose density gradients after endocytosis

Subconfluent cells were carefully washed in cold Ca^2+^/Mg^2+^-PBS, and lysed for 30 min on ice in ice-cold buffer A (150 mM NaCl, 2 mM EDTA, 50 mM Tris-HCl, pH 7.5) containing 1% Triton X-100. Cell lysates were centrifuged at 4°C for 5 min at 1200×g and sucrose density gradients were performed as described before. uPAR was immunoprecipitated from the different fractions in order to analyze the amount of receptor internalized. For immunoprecipitation, lysates were pre-incubated at 4°C for 2 h with protein A-sepharose beads, and then the supernatants were incubated overnight with rabbit anti-uPAR antibodies that had been previously bound to protein A-sepharose beads. Beads were then washed twice in lysis buffer, and eluted in non-reducing sample buffer. Samples were analyzed by 10% SDS-PAGE and transferred onto PVDF. The membrane was blocked in 5% BSA overnight and incubated with HRP-streptavidin (to identify the internalized fraction of uPAR).

### Biotinylation assay of uPAR endocytosis

Subconfluent cells were washed with Ca^2+^/Mg^2+^-PBS, and acid-treated in 50 mM Glycine hydrochloride/100 mM NaCl, pH 3, for 3 minutes at 0°C (to remove endogenously bound ligands), and quickly neutralized with a half volume of 0.5 M Hepes/100 mM NaCl, pH 7.4. Then, cells were incubated 1 h on ice in the presence of 0.5 mg/mL NHS-SS-biotin in PBS. Acid treatment efficiently eliminates any ligand bound to uPAR as demonstrated in [11]. Biotinylated cells were then washed with PBS and incubated at 37°C to allow internalization. The samples were then put back on ice, washed and treated with two successive reductions of 20 min on ice with a reducing solution containing 42 mM GSH, 75 mM NaCl, 1 mM EDTA, 1% BSA and 75 mM NaOH. In this way, only the internalized proteins remained biotinylated. Cells were then carefully washed in cold PBS, and lysed with RIPA buffer (50 mM Tris pH 7,6, 150 mM NaCl, 1% deoxycolate, 0.1% SDS, 1% Triton X-100) for 30 min. on ice. uPAR was immunoprecipitated from lysates to analyze the amount of receptor internalized. Peroxidase reaction was developed using ECL. A sample that was labelled but not reduced was included as a positive control, and a sample that was labelled and reduced, prior to incubation at 37°C, was included as a negative control. In order to normalize the internalized fraction of uPAR with respect to total uPAR, the membranes were stripped with stripping buffer, blocked on 5% non-fat milk, and then incubated with the monoclonal anti uPAR antibody R2. Band intensity was quantified by densitometry. To study endocytosis in cells treated with drugs, HEK293-uPAR cells were pre-incubated in medium containing the drugs at 37°C, followed by biotinylation at 0°C and internalization at 37°C. We used the following drugs: Wortmannin 300 nM for 1 hour, Amiloride 5 mM for 30 minutes. The inhibitors were also added during the internalization process. In other experiments, uPA:PAI-1 complexes were used. In this case, cells were biotinylated and then incubated 1h at 4°C with uPA:PAI-1 50 nM. Then, cells were washed and transferred to 37°C to allow internalization. In order to inhibit uPA:PAI-1-dependent endocytosis, 200 nM RAP was used. For experiments with RhoA, Rac-1 and cdc42 GDP-bound mutants, HEK293-uPAR cells were cultured on glass coverslips and transiently transfected with Fugene 6 (Roche) for 24 hours with 1 µg of the indicated DNA construct, and analyzed as described above.

### Recycling (biotinylation assay)

To follow uPAR recycling to the cell surface, HEK293-uPAR cells were biotinylated as mentioned above and incubated at 37°C for 20 minutes to allow internalization. Then cells were treated with GSH. The amount of uPAR that was internalized in this first step was considered as 100% of the internalized receptor for quantification purposes. Then cells were incubated again (in duplicates) at 37°C for 10 or 20 minutes to allow the recycling of the biotinylated (internalized) receptor to the cell surface. Next, one set of samples was treated with GSH, to obtain the amount of uPAR that recycled back to the cell surface. The other set of samples was kept with PBS instead of GSH, to obtain the amount of receptor that was either degraded or shed from the cell surface. Samples were collected and subjected to cell lysis, immunoprecipitation and immunoblotting as explained above. Recycling and degradation were calculated by subtracting the densitometric values of the residual biotinylated receptor, following incubation at 37°C and treatment with either GSH (recycling) or PBS (degradation) from the total pool of internalized receptor for 20 minutes at 37°C.

### Constitutive endocytosis (cell cytometry assay)

HEK293-uPAR cells were grown in 24-well plates up to 80% confluence and were first incubated at 0°C with a biotinylated polyclonal anti-uPAR antibody, to label the cell surface receptor. Then cells were incubated at 0°C with Neutravidin-Phycoerythrin (to label biotinylated antibodies). As control for autofluorescence, cells were only incubated with Neutravidin-Phycoerythrin. One set of samples was kept at 0°C while another set was transferred for 30 minutes at 37°C to allow internalization. Half of the samples kept at either 0 or 37°C were acid washed at 0°C to remove the surface-bound antibodies (AW). In this way, only the uPAR-bound internalized antibodies remain labeled. The other half of the samples were kept at 0°C, corresponding to total amount of labeled receptor at the cell surface. Then cells were analyzed by cell cytometry using a FACSCalibur cytometer (Beckton and Dickinson). Median fluorescence (MF) values were obtained that correspond to the amount of labeled receptor present in each set of samples. The amount of internalized receptor was calculated by subtracting the internalized fraction (IF) at 4°C from the internalized fraction at 37°C. The IF at 4 and 37°C were calculated according to:
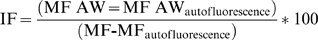



Other molecules tested with this assay were LRP-1, CD59, Transferrin, 70kDa-Dextran, and uPA:PAI-1 complexes. When amiloride was used, cells were pre-incubated with amiloride for 30 minutes at 37°C, followed by receptor labeling at 0°C, and internalization at 37°C in the presence of amiloride. For experiments with RhoA, Rac1 and cdc42 GDP-bound mutants, HEK293-uPAR cells were cultured on glass coverslips and transiently transfected with Fugene 6 (Roche) for 24 hours with 1 µg of the indicated DNA construct, and analyzed as described above but gating only GFP positive cells.

### Electron microscopy

#### Uptake of 5 nm gold BSA

After washing cells with serum-free DMEM, HT1080 cells were incubated for 2 hours at 37°C with BSA gold (*OD*_520 nm_ = 5) and processed as described below.

#### Cryosectioning and immunolabeling

According to the conventional protocol of Tokuyasu [41] adapted by Geuze and Slot [42], HT1080 cells were fixed with 2% paraformaldeyde and 0.2% glutaraldeyde in PBS for 2 hours at room temperature, embedded in 12% gelatin and cooled at 4°C for 10 minutes. Convenient blocks were cut and infiltrated in 2.3M Sucrose overnight and then frozen in liquid nitrogen. Ultrathin cryosections (60 nm) were cut and picked up in 1∶1 2.3M Sucrose and 2% Metylcellulose. Cryosections were immediately immunolabeled for uPAR with anti-uPAR antibodies and detected with Protein A gold 10 nm as previously described [42].

#### Endocytic assay

For embedding in epoxy resin (Epon LX112) the cells were grown to 80% confluence, and cultured in normal medium or with 200 nM RAP for 1 hour at 37°C, when indicated. Subsequently, cells were washed twice with ice cold HBSS medium and incubated with the primary antibody (monoclonal R3 antibody, anti-human uPAR) alone or with 200 nM RAP for 1 hour at 0°C. Cells were washed with pre-chilled HBSS medium to remove the excess of the antibody and incubated with a rabbit anti-mouse (Jackson, dilution 1∶100) for 30 minutes at 0°C. After extensive washing, cells were incubated with Protein A gold 10 nm (Utrecht, The Netherlands, dilution 1∶80) for 30 minutes at 0°C. Cells were re-washed and incubated with pre-warmed HBSS for different time (0 minutes, 5 minutes and 20 minutes) at 37°C in order to allow internalization. The RAP inhibitor was also used during endocytosis. The cells were then processed for conventional electron microscopy. Briefly, after fixation in 2,5% glutaraldeyde in 0.1 M Sodium Cacodylate buffer for 10 minutes at room temperature, cells were gently scraped from petri dishes and washed 5 minutes with 0.1 M Sodium Cacodylate buffer. Upon centrifugation, pellets were post-fixed in 1% Osmium Tetroxide diluted in 0.1 M Sodium Cacodylate buffer for 10 minutes at room temperature and washed with distilled water. Then, staining for 1 hour at room temperature with 1% Uranyl Acetate was performed. Subsequently, the pellets were dehydrated through a graded ethanol series and embedded in LX112 resin overnight at 42°C and 2 days at 60°C. Ultrathin plastic sections of 50 nm were stained with Uranyl Acetate and Lead Citrate and viewed using either a Philips CM10 or a Tecnai G2 (FEI Company) electron microscope.
